# Cambios en la epidemiología de la tosferina en Málaga (2017-2024)

**DOI:** 10.23938/ASSN.1091

**Published:** 2024-12-05

**Authors:** Ignacio Rodríguez-Vergara Pérez, David Moscoso Sánchez, Julián Manuel Domínguez Fernández, Sara Estefanía Montenegro Jaramillo, María Amores Alguacil

**Affiliations:** 1 Hospital Universitario de Ceuta Servicio de Medicina Preventiva y Prevención de Riesgos Laborales Ceuta España; 2 Servicio Andaluz de Salud Delegación Territorial de Salud y Consumo en Málaga Sección de Epidemiología Málaga España

**Keywords:** Tosferina, Hospitalización, Vacunación, Notificación de Enfermedades, Vigilancia en Salud Pública, Whooping Cough, Hospitalization, Vaccination, Disease Notification, Public Health Surveillance

## Abstract

**Fundamento:**

Describir la evolución epidemiológica de la tosferina en la provincia de Málaga en el periodo 2017-2024, la gravedad de la enfermedad y su vacunación.

**Metodología:**

Estudio transversal de casos de tosferina declarados obtenidos de la red de notificación de Enfermedades de Declaración Obligatoria de Andalucía (RedAlerta). Se calculó el número de casos por 100.000 habitantes por distrito sanitario, trimestre y año. Se analizó la relación entre hospitalizaciones y estado vacunal.

**Resultados:**

Se notificaron 181 casos de tosferina en el periodo de estudio, el 56,4% durante el periodo pre-pandémico (2017-2019) y solo el 9,95% durante 2020-2023. En el primer trimestre de 2024 aumentaron los casos notificados (n = 61; 33,7% del total) respecto a los años previos (1-46 casos). El grupo de 0 a 20 años supuso el 71,3% de los casos, y el 57,4% había recibido al menos la pauta parcial de vacunación antes de sufrir tosferina. El 25,6% de los casos requirieron ingreso, más frecuente entre los no vacunados (70 frente a 18,3%; p<0,001); el 42,4% no tenían administrada ninguna dosis previa. El 47,1% de los 17 casos menores de 1 año no habían recibido ninguna dosis ni tenían antecedentes de vacunación materna frente a tosferina durante el embarazo.

**Conclusiones:**

En el primer trimestre de 2024 se detectó un aumento de casos declarados de tosferina en Málaga, mientras que los casos hospitalizados se mantuvieron estables. Se detectó una relación significativa entre la vacunación previa frente a tosferina y su gravedad (hospitalización) en el grupo de 0 a 20 años.

## Abstract (en)

**Background:**

Description of the epidemiological evolution of pertussis in the province of Malaga, Spain (2017-2024), disease severity, and vaccination coverage.

**Methods:**

A cross-sectional study was conducted using reported cases from the Andalusian Notifiable Diseases Network (Red Alerta). The incidence of pertussis was calculated per 100,000 inhabitants for each health district, quarter, and year. Additionally, the relationship between severe cases (hospitalizations) and vaccination status was analyzed.

**Results:**

A total of 181 pertussis cases were identified. Of these, 56.4% were reported during the pre-pandemic period (2017-2019), while 9.95% occurred between 2020 and 2023. An increase in cases was observed in the first quarter of 2024 (n = 61; 33.7% of the total cases), compared to previous years (1 to 46 cases). The 0 to 20-year group accounted for 71.3% of cases, of which 57.4% had received at least partial vaccination prior to contracting pertussis. Severe disease requiring hospitalization occurred in 25.6% of cases, with a higher frequently among unvaccinated individuals (70% vs 18.3%; p < 0.001). Among hospitalized cases, 42.4% had not received any prior doses of the vaccine. Additionally, 47.1% of the 17 pertussis cases in children aged ≤1 year had not received any vaccination and had no history of maternal vaccination during pregnancy.

**Conclusions:**

An increase in reported pertussis cases is observed in early 2024 in Malaga, but hospitalization rates among confirmed cases remained stable. A significant association is found between prior vaccination against pertussis and reduced disease severity (hospitalization) in the 0 to 20-year-old age group.

## INTRODUCCIÓN

La infección por bacterias del género *Bordetella,*especialmente las especies *pertussis*y parapertussis, causa tosferina^1^^,^^2^, una enfermedad muy contagiosa con una tasa de ataque secundario (TAS) superior al 80%^3^. La enfermedad se transmite a través del contacto directo con las secreciones respiratorias o con las gotículas de saliva de una persona infectada^4^. 

La OMS estima que en 2014 hubo 24,1 millones de casos y 160.700 muertes por la enfermedad en niños menores de 5 años en el mundo^5^. En España, la incidencia en los primeros meses de 2024 ascendió a 81,2 casos/100.000 habitantes, mientras que en 2023 se registraron 5,78 casos/100.000 habitantes^6^; sin embargo, el porcentaje de casos hospitalizados entre enero y abril de 2024 en España es un 1,34% menor que en 2023^6^. 

Aunque los últimos resultados de seroprevalencia indican que la circulación de *Bordetella pertussis*ocurre en todos los grupos de edad^7^, las tasas de casos declarados son clásicamente más elevadas en menores de 1 año. Estos pacientes que son más susceptibles de sufrir formas graves de tosferina^3^, también conocidas como tosferina maligna, que pueden producir hipertensión pulmonar, hiperleucocitosis e hipoxemia refractaria, todos ellos signos de mal pronóstico^8^.

La vacunación disponible para la tosferina consiste en una vacuna acelular completa contra *B. pertussis*junto con los toxoides contra la difteria y el tétanos^9^. La aplicación masiva de esta vacuna a nivel mundial redujo la tasa de infección por 100.000 habitantes de 157 a menos de 1 en la década de los 70^10^. Actualmente, el calendario de vacunación infantil en Andalucía (España), y por tanto en Málaga, incluye la vacunación frente a la tosferina con una serie primaria de dos dosis a los 2 y 4 meses de edad, y dos dosis de refuerzo a los 11 meses y a los 6 años^11^(hasta 2016 se administraba una dosis adicional a los 6 meses, eliminada con la modificación del calendario en 2017). Además, desde 2015 se vacuna a las mujeres embarazadas entre las semanas 28 y 36 de gestación, para prevenir la tosferina en menores de 3 meses de edad^11^. En Andalucía, a día de hoy, la cobertura vacunal de la tosferina en menores casi alcanza el 95%, y la vacunación de las gestantes supera el 90%^11^.

La tosferina se comporta de forma cíclica en España, donde el último periodo epidémico registrado fue en la temporada 2014-2015^12^. Estos ciclos epidémicos habitualmente abarcan entre tres y cinco años^13^, pudiendo variar entre las diferentes provincias del país.

La provincia de Málaga está dividida en seis distritos sanitarios: Axarquía, Costa del Sol, La Vega, Málaga, Serranía y Valle del Guadalhorce^14^. Cada distrito se encuentra compuesto por las denominadas zonas básicas de salud que engloban a los diferentes municipios que forman parte de su área^14^.

El objetivo de este estudio es describir la evolución epidemiológica de la tosferina en Málaga desde el año 2017 hasta el primer trimestre de 2024, analizar la variación en la gravedad de la enfermedad, así como evaluar la situación de la vacunación en 2024. Todo ello nos ayudaría a comprender la situación actual de la enfermedad y su posible relación con la pandemia por COVID-19, para poder abordar los debates sobre la necesidad de tomar medidas poblacionales acordes a los datos más actualizados. 

## MATERIAL Y MÉTODOS

Se realizó un estudio transversal de los casos de tosferina declarados entre el 1 de enero de 2017 y el 31 de marzo de 2024 en la provincia de Málaga (Andalucía, España). 

Los datos de los casos de tosferina declarados durante el periodo de estudio en la provincia de Málaga se extrajeron de la red de notificación de Enfermedades de Declaración Obligatoria (EDO) de Andalucía (RedAlerta). 

Se tuvieron en cuenta las siguientes consideraciones^4^:

- Entre 2017 y 2023 se incluyeron todos los casos registrados clasificados como 1) *Confirmado*: personas que cumplen los criterios de laboratorio (aislamiento de *B. pertussis*o detección de su ácido nucleico en una muestra clínica, o detección de anticuerpos específicos frente a *B. pertussis*); 2) *Sospechoso*: personas que cumplen los criterios clínicos (tos paroxística, estridor inspiratorio o vómitos provocados por la tos durante, al menos, dos semanas; diagnóstico de tosferina por un profesional médico; y menores de un año con episodios de apnea); 3) *Probable*: personas que cumplen criterios clínicos y tienen un vínculo epidemiológico. No se consideraron los casos clasificados como *descartados*.
- En los primeros tres meses de 2024, desde la Delegación Territorial de Salud y Consumo se solicitó a los distintos Distritos Sanitarios de Málaga que revisaran los casos que figuraban como *sospechosos*en la plataforma de notificación a fecha de abril de 2024 y que los modificaran de acuerdo a si habían sido confirmados o descartados mediante pruebas de laboratorio, a fin de trabajar únicamente con datos de casos confirmados, ya que los casos registrados como sospechosos superaban ampliamente a los registrados como confirmados. Este proceso no se realizó entre 2017 y 2023 porque el número de casos registrados como sospechosos en ese periodo de tiempo era insignificante y contaban con una elevada sospecha diagnóstica.

Para calcular el número de casos/100.000 habitantes y año se dividió el número de casos de tosferina al final de cada año entre el número de habitantes, y el resultado se multiplicó por 100.000. Se realizaron estos cálculos en la provincia y por cada Distrito de Salud según los datos del padrón de habitantes a 1 de enero de cada año anterior al año estudiado; el número de habitantes osciló entre 1.629.298 en 2016 y 1.752.728 en 2023. Con estos datos se realizó la curva epidemiológica de la enfermedad. 

Se registraron las siguientes variables de pacientes: sexo (femenino, masculino), edad (0 a 20 años, 21 a 65, ≥66 años), país de origen, trimestre de declaración, ingreso hospitalario, y coordenadas geográficas del domicilio de los casos identificados. Además, se crearon tres subgrupos dentro de la categoría 0 a 20 años para diferenciar entre los posibles estados de vacunación de las personas según el calendario vacunal establecido: ≤1 año (sin vacunación o vacunación parcial), 2 a 6 años (vacunación que puede incluir hasta la última dosis de recuerdo) y 7 a 20 años (vacunación completa). 

Se analizó la situación vacunal previa a la infección por *B. pertussis*en el periodo de estudio, incluyendo el número de dosis aplicadas y fecha de las mismas, además del antecedente de vacunación materna durante el embarazo. La información vacunal se obtuvo directamente de las historias clínicas en el programa Diraya.

Este estudio recibió el visto bueno por parte de la Comisión de Formación Continuada de Docencia e investigación del Hospital Universitario de Ceuta, y por parte de los jefes de Salud y de la Sección de Epidemiología de la Delegación Territorial de Salud y Consumo de la provincia de Málaga.

### Análisis estadístico

Se utilizaron bases de datos de elaboración propia con el *software Open Office*y el cálculo de los estadísticos se realizó con el *software*de libre uso *Epi Info*(versión 7.2.6.0). 

La descripción de las variables cualitativas se realizó con frecuencias y porcentajes, y de las variables cuantitativas con la media y la desviación estándar (DE), tanto para el conjunto completo de datos como para cada año individualmente y para los tres primeros meses de cada año.

Para determinar si existían diferencias significativas entre variables de interés se emplearon la prueba de Ji-Cuadrado (**χ^2^**) para las variables cualitativas y el test de Kruskal-Wallis para las cuantitativas. El nivel de significación estadística se estableció en p<0,05.

También se utilizó el *software*QGIS para crear un mapa con la localización de los casos de tosferina, a partir de las coordenadas X e Y del domicilio de cada caso.

## RESULTADOS

**Figura 1:**
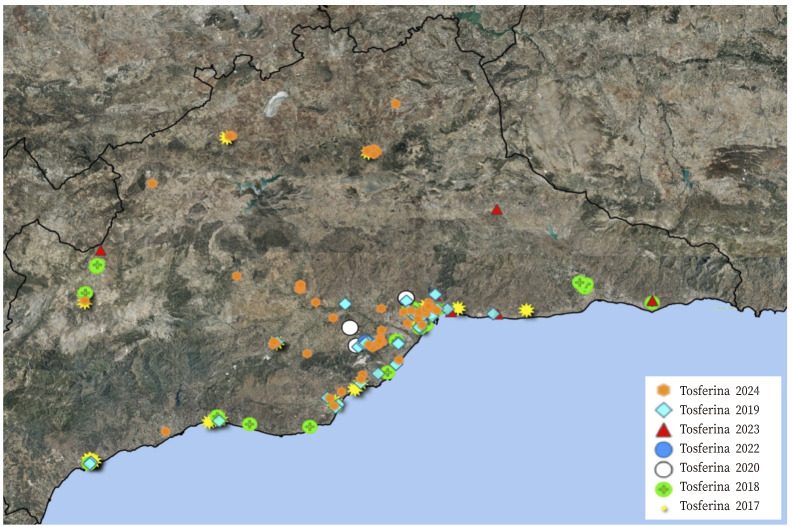
Mapa de los casos de tosferina declarados en la provincia de Málaga de 2017 a 2024.

En el periodo de estudio se registraron 181 casos de tosferina (Fig. 1). El 33,70% de los casos correspondieron a los declarados en los tres primeros meses de 2024, el 56,35% correspondieron al periodo pre-pandémico (2017-2019) y el 9,95% restante se declararon durante el periodo pandémico por SARS-CoV-2. No se registró ningún caso *probable*.

En el primer trimestre de 2024 se observó un aumento del número de casos de tosferina declarados, tanto a nivel provincial como en cada distrito sanitario, respecto al primer trimestre de cada año anterior, exceptuando el distrito Axarquía que no registró ningún caso en este trimestre de 2024 (Tabla 1). Los trimestres segundo o tercero de cada año completo dentro del periodo de estudio fueron los que acumularon más casos (Tabla 1).

**Tabla 1:** Casos de tosferina por 100.000 habitantes declarados en la provincia de Málaga entre 2017 y 2024

	2017	2018	2019	2020	2022	2023	2024	Total
							T1	n (%)
*Casos por distrito sanitario*								
Axarquía								
Anual	1	5	0	0	0	4	0	10 (5,5)
T1	0	1	0	0	0	0	0	1 (1,2)
Costa del Sol								
Anual	13	9	12	0	0	0	7	41 (22,6)
T1	5	1	2	0	0	0	7	15 (18,5)
La Vega								
Anual	2	0	0	0	0	0	18	20 (11,1)
T1	1	0	0	0	0	0	18	19 (23,5)
Málaga								
Anual	6	11	19	3	0	5	13	57 (31,5)
T1	0	1	2	0	0	0	13	16 (19,8)
Serranía								
Anual	3	4	0	0	0	1	1	9 (5,0)
T1	0	0	0	0	0	0	1	1 (1,2)
Valle del Guadalhorce								
Anual	2	0	15	2	1	2	22	44 (24,3)
T1	2	0	3	0	0	2	22	29 (35,8)
*Provincia*								
Anual	27	29	46	5	1	12	61	181
T1	8	3	7	0	0	2	61	81
*Casos por trimestre*								
T1	8	3	7	0	0	2	61	81 (44,8)
T2	3	10	14	4	0	1	-	32 (17,7)
T3	11	13	14	1	0	5	-	44 (24,3)
T4	5	3	11	0	1	4	-	24 (13,2)

DE: desviación estándar; T: trimestre.

**Figura 2:**
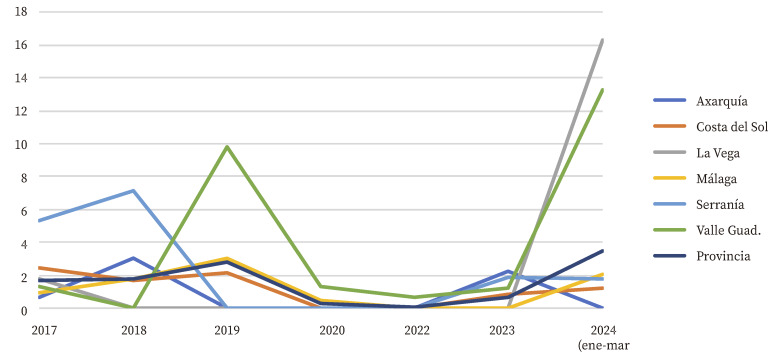
Casos de tosferina por 100.000 habitantes y por año en la provincia de Málaga y por distrito sanitario.

El número de casos/100.000 habitantes registrados en cada distrito sanitario tendió a mantenerse e incluso a reducirse en el periodo 2017-2020. Sin embargo, se observó un aumento de los mismos en el primer trimestre de 2024 que llegó a igualar o incluso superar a los años anteriores completos para cada mismo distrito (Fig. 2). Este aumento fue mucho más acentuado en La Vega y Valle del Guadalhorce, que a pesar de ser los dos distritos sanitarios menos poblados de toda la provincia, después de Serranía, registraron el mayor número de casos en el primer trimestre de 2024 (Tabla 1).

Las personas con tosferina incluidas en el estudio mostraron un ligero predominio del sexo masculino (50,83%), con media de edad 16,80 años (DE = 20,70) y gran parte de los casos englobados en el grupo de 0 a 20 años (71,27%). Además, el 95,58% de los casos fueron de nacionalidad española (Tabla 2). En 2017, primer año del periodo de estudio, la hospitalización de los casos declarados fue superior (25,93%) que el resto de años (16-20%). La edad media de los hospitalizados fue 4,37 años, mayormente pacientes con 1 año o menos (n = 26; 74,29%) (Tabla 2).

**Tabla 2:** Características sociodemográficas de los casos de tosferina declarados en la provincia de Málaga entre 2017 y 2024

	2017	2018	2019	2020	2022	2023	2024	Total
							T1	
Edad, media (DE)	13	20,34	24,19	22,8	22	11,42	11,29	16,80 (20,70)
	-23,21	-21,89	-22,17	-22,15	(-)	-18,22	-16,04	
*Grupo de edad, n (%)*								
0 - 20	22 (17,1)	18 (14,0)	23 (17,8)	3 (2,3)	0	10 (7,8)	53 (41,1)	129 (71,3)
≤1	14	10	11	1	0	3	10	49
2-6	3	2	6	0	0	6	25	42
7-20	5	6	6	2	0	1	18	38
21 - 65	3 (6,4)	10 (21,3)	22 (46,8)	2 (4,3)	1 (2,1)	2 (4,3)	7 (14,9)	47 (26,0)
≥ 66	2 (40,0)	1 (20,0)	1 (20,0)	0	0	0	1 (20,0)	5 (2,7)
*Sexo, n (%)*								
Hombre	15 (16,3)	14 (15,2)	22 (23,9)	2 (2,2)	1 (1,1)	6 (6,5)	32 (34,8)	92 (50,8)
Mujer	12 (13,5)	15 (16,9)	24(27,0)	3 (3,4)	0 (0,0)	6 (6,7)	29 (32,6)	89 (49,2)
*Nacionalidad, n (%)*								
Española	26 (15,0)	26 (29,2)	43 (48,3)	5 (5,6)	1 (1,1)	12 (13,5)	60 (67,4)	173 (95,6)
Otra	1 (12,5)	3 (37,5)	3 (37,5)	0 (0,0)	0 (0,0)	0 (0,0)	1 (12,5)	8 (4,4)
*Hospitalización*								
n (%)	7 (25,93)	5 (17,24)	8 (17,39)	1 (20)	0	2 (16,67)	12 (19,67)	35 (19,34)
*Por grupo de edad*								
≤1	7	5	6	1	0	0	7	26 (74,3)
2-6	0	0	1	0	0	2	3	6 (17,1)
7-20	0	0	0	0	0	0	1	1 (2,9)
21-65	0	0	1	0	0	0	0	1 (2,9)
≥ 66	0	0	0	0	0	0	1	1 (2,9)

DE: desviación estándar; T: trimestre.

Solo se consiguieron los datos de antecedentes de vacunación frente a *B. pertussis*en el grupo de 0 a 20 años de edad (n = 129), ya que las historias clínicas del resto de grupos etarios no disponían de estos datos. Más de la mitad de pacientes de este grupo había recibido la pauta parcial de vacunación (57,36%) antes de sufrir tosferina. Una cuarta parte de los casos (25,6%) requirieron ingreso, más frecuentemente entre los casos no vacunados (70% frente a 18,3%; p<0,001; X^2^). Considerando los 33 casos (25,6%) que requirieron ingreso hospitalario, el 42,42% no tenían administrada ninguna dosis previa, y el 88,90% de los que no requirieron hospitalización habían recibido al menos una dosis de vacuna antes de ser contagiados (Tabla 3). En el subgrupo de edad ≤1 año hubo 17 casos que no recibieron ninguna dosis de vacuna antes del evento (34,7%), y las madres de 8 de estos 17 casos rechazaron la vacunación durante el embarazo, mientras que 9 la aceptaron.

**Tabla 3:** Vacunación en el grupo de 0 a 20 años de edad antes de sufrir tosferina durante el periodo 2017-2024, y frecuencia de hospitalización según el estado de vacunación

	n (%)	Hospitalización (n = 33)
*Vacunación*		
completa	30 (23,26)	19 (18,27)
parcial	74 (57,36)	
no iniciada	20 (15,50)	14 (70,0)
sin datos	5 (3,83)	0

## DISCUSIÓN

Nuestros resultados apuntan a que la pandemia por COVID-19 pudo propiciar el escenario al que hoy nos enfrentamos, con un gran aumento de casos de tosferina declarados en el primer trimestre de 2024 en Málaga. Las restricciones aplicadas a nivel global para frenar la circulación del SARS-COV-2 y para combatir la COVID-19^15^habrían cortado de forma muy contundente la exposición de la población general a *B. pertussis*y a *B. parapertussis*. La circulación limitada de *Bordetella*podría haber coincidido con el repunte periódico esperable de tosferina. Ahora nos encontramos en un contexto de post-pandemia en el cual se han retirado todas esas medidas restrictivas de contacto personal, lo que es posible que haya propiciado el aumento del flujo de *Bordetella*.

Otro efecto de la pandemia fue el desarrollo de pruebas diagnósticas enfocadas a combatirla, especialmente la detección de ácido nucleico por reacción en cadena de la polimerasa (PCR)^4^y los cultivos bacterianos en medios específicos, como consecuencia del cual las pruebas son más sensibles y específicas, lo que agiliza la identificación de casos^16^y ha aumentado la confirmación de los mismos.

Además, varios artículos en la prensa local de la provincia podrían haber provocado un aumento de la demanda de atención sanitaria en casos sospechosos de tosferina, que en condiciones normales probablemente no habrían consultado, y que se acabaron confirmando con pruebas de laboratorio.

Esta mayor notificación de casos en el primer trimestre de 2024 respecto al mismo trimestre de años anteriores aumenta la probabilidad de que se registre un aumento muy marcado del número de casos por 100.000 habitantes a final de año respecto a los años anteriores. Aunque los meses de diciembre a marzo se han descrito como los de mayor aparición de casos de tosferina en España^17^, nuestros resultados apuntan a que, al menos desde 2017 en Málaga, los meses de mayor transmisibilidad de la enfermedad suelen ser los comprendidos entre el segundo y tercer trimestre. Por tanto, si se mantiene la tendencia de los últimos años, podemos esperar un aumento del número de casos declarados en los meses de abril a septiembre y, por ende, del total de caso/100.000 habitantes de la provincia a final de año.

Aunque el aumento del número total de casos hace que la frecuencia absoluta de hospitalizaciones también aumente, el porcentaje de hospitalizaciones respecto al total de casos se mantiene dentro de los límites usuales de otros años, lo que indica que la gravedad de la enfermedad sigue estando controlada gracias a las tasas de vacunación elevadas. La vacunación es un pilar fundamental para evitar los casos graves de enfermedad y la consecuente hospitalización, tal y como muestran nuestros resultados en concordancia con otros estudios publicados^18^^,^^19^^,^^20^.

La vigilancia hospitalaria exhaustiva a través de pruebas serológicas de los casos sospechosos de tosferina que requieren ingreso es una opción a considerar en casos de ciclos epidémicos^21^, por varios motivos. Ayudaría a estimar a situación real poblacional de la enfermedad, ya que la dimensión de la situación hospitalaria se relaciona clásicamente con la punta de un iceberg^22^^,^^23^, en el que la situación poblacional es la parte del iceberg que no se deja ver y que podemos esperar que tenga una dimensión mayor o menor dependiendo de la dimensión de la situación hospitalaria, situación que sí vemos y que tenemos bien controlada gracias a la serovigilancia de estos casos más graves. También contribuiría a mejorar los servicios brindados, ya que la colaboración entre los cuidados clínicos y la salud pública puede mejorar la entrega de servicios sanitarios, abordando problemas de salud comunitarios y aplicando una perspectiva poblacional a la práctica clínica^24^. 

Respecto a la gravedad de la tosferina durante los primeros meses de vida, la vacunación en el embarazo es una medida efectiva para reducir la morbimortalidad en niños por debajo de seis meses de edad^25^^,^^26^. Nuestros resultados no han podido demostrar de forma concluyente este aspecto debido al bajo número de niños hospitalizados sin antecedente previo de vacunación antes de la infección por *Bordetella*, pero sí se ha visto que casi la mitad de los niños de hasta un año que contrajeron tosferina durante el periodo de estudio no tenían ninguna dosis de vacuna administrada, ni tampoco antecedentes de vacunación materna durante la gestación.

No haber tenido en cuenta los datos de casos sospechosos del primer trimestre de 2024 implica que el número de casos por 100.000 habitantes reportado es menor al real, lo que podría subestimar el impacto total de la enfermedad en la población, y que asumimos como principal limitación de este estudio. A consecuencia de ella, si el número de casos reales de tosferina en el primer trimestre de 2024 es mayor al observado, la proporción de casos hospitalizados disminuiría de forma considerable, reforzando nuestras afirmaciones acerca de que la gravedad y hospitalización de los casos se encuentran dentro de los rangos habituales de los últimos años y; por lo tanto, controladas. Además, la disponibilidad de pruebas de tosferina en hospitales facilita mucho la identificación de casos y hace bastante improbable que no se confirme un caso de tosferina hospitalizado, por lo que estos casos hospitalizados sí reflejan la situación real por la que están pasando los hospitales. Otras limitaciones podrían ser el pequeño tamaño de muestra de algunos grupos, que ha reducido la relevancia de los resultados obtenidos, así como el tipo de estudio realizado, que al centrarse en un periodo de estudio de casi ocho años en una provincia concreta podría limitar la validez externa del propio estudio.

En conclusión, en el primer trimestre de 2024 se ha detectado un aumento de casos declarados de tosferina en Málaga, mientras que el porcentaje de casos hospitalizados se mantiene en niveles estables. 

Consideramos primordial controlar la gravedad de la infección por tosferina mediante la vacunación, incentivándola en las consultas de Atención Primaria para aumentar su aceptación en la población general. Así se lograría mantener niveles elevados tanto de vacunación infantil como de gestantes, protegiendo a la población infantil de contraer formas graves de tosferina a edades tempranas. A fin de mantener bajo control el indicador de la gravedad de la tosferina, y si la labor de los epidemiólogos de campo se viera desbordada por el aumento de casos comunitarios de menor gravedad, sugerimos concentrar los esfuerzos en la situación hospitalaria de la enfermedad, convirtiendo la declaración de casos hospitalizados en el trabajo prioritario de los profesionales de la salud pública.

## Data Availability

Se encuentran disponibles bajo petición al autor de correspondencia.
